# Physical activity and persistent low back pain and pelvic pain post partum

**DOI:** 10.1186/1471-2458-8-417

**Published:** 2008-12-22

**Authors:** Ingrid M Mogren

**Affiliations:** 1 Department of Clinical Science, Obstetrics and Gynaecology, Umeå University, Umeå, Sweden; 2 Department of Public Health and Clinical Medicine, Epidemiology and Public Health Sciences, Umeå University, Umeå, Sweden

## Abstract

**Background:**

The aims of this study were (i) to investigate the potential influence of pre-pregnancy regular leisure-time physical activity (PA) on the risk of persistent LBPP half a year after pregnancy, and (ii) to explore the starting time and prevalence of PA among women experiencing LBPP during pregnancy, in relation to remission or persistent LBPP half a year after pregnancy.

**Methods:**

This study is a follow-up study of 639 women who reported LBPP during pregnancy. These women were sent a questionnaire at approximately six months after delivery. The respondents were divided into three groups: 'no pain', 'recurrent pain', and 'continuous pain'. Data were analysed using an independent samples t-test, Pearson's chi-squared test, and univariate and multivariate logistic regression analyses.

**Results:**

44.5% of subjects reported current PA at six months post partum. The mean starting time of PA was 2.6 months post partum and the mean number of current, weekly events of PA was 3.4; there were no differences between the groups. 82.2% reported previous PA at some period in life. Women with BMI ≥ 30 reported current PA to a lesser extent. The number of years of pre-pregnancy PA did not influence the risk of persistent LBPP.

**Conclusion:**

Almost half of women who had experienced LBPP during pregnancy reported PA at six months post partum. The number of years of pre-pregnancy PA did not influence the risk of persistent LBPP. Obesity was a risk factor for not practising PA.

## Background

A majority of women suffer from low back pain and pelvic pain during pregnancy [1,2]. This pregnancy-related condition negatively influences self-estimated health and interferes with sexual activity during pregnancy [3]. The risk of experiencing LBPP in a subsequent pregnancy is extremely high [4,5]. Around four out of ten women report persistent LBPP half a year after delivery [6-9]. Remission of the condition primarily occurs during the first six months after delivery [10]. At three years post partum, 20% of all women with back pain during pregnancy report persistent symptoms [11]. Post partum back pain has been associated with considerable perceived disability in movement-related activities [12].

Physical activity is a major determinant of life-long health [13,14], and is well known to be beneficial for physical and psychological well-being before, during, and after pregnancy [15,16]. Unfortunately, the epidemiology of physical activity shows a consistent decline from adolescence to young adulthood [14]. Physical activity usually declines during pregnancy [17,18], but increased well-being has been found in women who maintain or increase their levels of exercise and sporting activity post partum [19]. In an American study, fitness and strength declined relative to pre-pregnancy levels in the early post partum period, but improved by 27 weeks post partum [20]. We have previously demonstrated that a higher number of years of regular leisure-time physical activity (PA) prior to pregnancy decreases the risk of LBPP during pregnancy [21].

The aetiology of LBPP is still poorly understood [22]. Further, there is as yet no consensus over the definition of the condition, although attempts have been made [22,23]. Results from the present cohort have previously been reported [3,5,9,21,24], with the prevalence of persistent LBPP after pregnancy being estimated at 43% [9].

The aims of this study were (i) to investigate the potential influence of pre-pregnancy PA on the risk of persistent LBPP half a year after pregnancy, and (ii) to explore the starting time and prevalence of PA among women experiencing LBPP during pregnancy, in relation to remission or persistent LBPP half a year after pregnancy.

To our knowledge there are currently no publications that have addressed these research questions.

The study was approved by the Ethics Committee at Umeå University (Dnr. 01-335). Eligible subjects were informed and respondents participated after informed consent.

## Methods

The study population was drawn from that of a previous study [5], in which all women who gave birth between 1 January 2002 and 30 April 2002 in the Departments of Obstetrics and Gynecology at Umeå University Hospital (UUH) and the Sunderby Hospital (SH) in the counties of Västerbotten and Norrbotten in northern Sweden were invited to participate. The women received verbal and written information on the aims of the study from a midwife on duty at the department, usually within 24 hours of delivery. Women who agreed to participate received a questionnaire (Q1) on their obstetric and gynaecological history, actual pregnancy, and delivery. Each questionnaire contained a unique number, and the identification numbers of the women who declined participation were likewise recorded, for analysis of missing data. For inclusion in the study, the women had to have reached a gestational age of at least 23 weeks, ending in a live birth or stillbirth. The study used a cross-sectional design. During the period of 1 January 2002 to 30 April 2002, the total number of women delivered at UUH and SH was 1114, with 516 (46.3%) delivered at UUH and 598 (53.7%) at SH.

Another inclusion criterion was competence in the Swedish language, which decreased the number of eligible women to 1071. Non-respondents were defined as women who either did not receive a questionnaire or did not complete the questionnaire they were given. The net sample consisted of 891 respondents (Q1), a response rate of 83.2%. Place of delivery did not influence risk in the logistic regression analyses. Detailed information on the sample has been presented in a previous paper [5].

Women reporting LBPP (n = 639) during pregnancy (Q1) were followed up with a second questionnaire (Q2) at approximately six months after delivery, thus constituting a cohort. The questionnaire included 39 questions on different issues such as LBPP after pregnancy, use of medical services, family situation, perceived health, sick leave, sexual activity, physical activity, oral contraception, and breast-feeding. One or more reminders were sent to those subjects who did not respond to the first request to complete Q2. Altogether 77.0% (n = 492) of the 639 eligible subjects responded to Q2. Twenty-eight women were excluded because they completed the questionnaire 9 months or more after date of delivery. The net sample therefore comprised 464 women (72.6% of eligible subjects).

### Definitions

*Low back pain or pelvic pain during pregnancy (LBPP) *in the previous study (Q1) was defined as 'recurrent or continuous pain for more than 1 week from the lumbar spine or pelvis' during recent pregnancy. A woman was considered to have LBPP during pregnancy if she gave a positive answer to the specific question on localisation of pain, which included marking the affected area on a drawing included in the questionnaire [5].

*Actual low back pain and pelvic pain (LBPP) after pregnancy *in the present study (Q2) was defined as a positive response to the question whether the subject had *actual *low back pain or pelvic pain. The response alternatives to this question were 'yes, recurrent pain', 'yes, continuous pain', and 'no pain'. Fourteen women gave a time point at which LBPP had ceased; however, they also declared that they had since had recurrent pain. These subjects were allocated to the 'no pain' group.

*Persistent LBPP after pregnancy *included women with both 'recurrent pain' and 'continuous pain' defined as LBPP after pregnancy.

*Body mass index (BMI) *was defined as weight (kg)/height^2 ^(m^2^).

### Statistics

Mean values and standard deviations (SD) were calculated for parametric data. An independent samples *t*-test and Pearson's chi-squared test were used to test the differences between two groups for parametric and categorical data, respectively. Odds ratios (OR) and their corresponding 95% confidence intervals (CI) were calculated by using logistic regression in univariate and multivariate analyses.

## Results

### Physical activity after pregnancy

**Figure 1:**
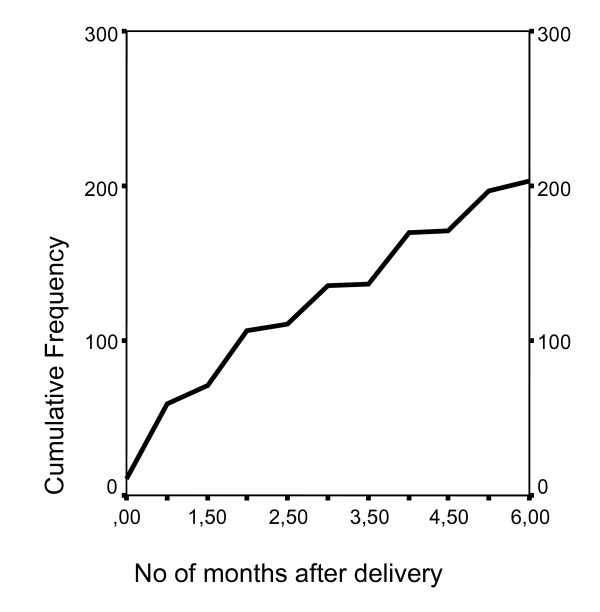
**Number of months after pregnancy at which PA was initiated or resumed**.

The proportion of women with current PA at approximately 6 months after delivery was 44.5% (Table 1, see additional file 1). There were no differences between the various sub-groups in either age at start of PA previously in life or prevalence of events of PA, mean number of events of PA, and starting time of PA after delivery (Table 1, see additional file 1). Figure 1 shows the starting time of PA after delivery for all respondents. Of the 378 women who reported PA at some period in their life prior to pregnancy, 48.9% (n = 185) reported current PA, while for the 82 who reported no PA prior to pregnancy, the corresponding figure was 23.2% (n = 19).

### Physical activity prior to pregnancy

Univariate and multivariate logistic regression analyses of the number of years of pre-pregnancy PA did not reveal any significant impact on the risk of persistent LBPP six months after delivery (Table 2, see additional file 2).

### BMI and physical activity

A higher proportion of women with BMI < 30 than women with BMI ≥ 30 reported current PA (Table 3, see additional file 3) (OR = 1.86; 95% CI = 1.01–3.42). The odds ratio was unaltered when adjusting for persistent LBPP (OR = 1.86; 95% CI = 1.01–3.43). There were no differences between these two groups in reported pre-pregnancy PA (some period in life), mean number of weekly events of current PA, and start of PA after delivery (Table 3, see additional file 3).

### Validity of the data, and non-respondent data

The validity of the data in Q1 has been extensively discussed in previous publications [3,5,9,21]. The sample of eligible women at delivery was 1071 women and the participation rate in Q1 was 83.2%. The non-respondents to Q1 were of the same age, had the same number of pregnancies and births, and were delivered by the same methods as the respondents [5]. Pre-term births and CS were more frequent among non-respondents to Q1 [5].

The eligible subjects in the present study were the 639 women who reported LBPP during pregnancy. The response rate to Q2 was 72.6% (n = 464). There were no statistically-significant differences (using a *t-*test or Pearson's chi-squared test as appropriate) between respondents and non-respondents with regard to age at start of PA, pre-pregnancy PA, maternal age, gestational age, birth weight, mode of delivery, onset of pain due to LBPP during pregnancy [9], family situation during pregnancy, relationship status before pregnancy, a satisfactory sexual life before or during pregnancy, and perceived health before and during pregnancy [3]. Non-respondents had had significantly more pregnancies and deliveries and a higher level of pain due to LBPP during pregnancy (Q1); this group also included a lower proportion than the respondent group of university-educated women [9].

## Discussion

Regular, non-excessive physical activity during pregnancy has been shown to have positive physiological effects on maternal and foetal outcomes [25]. However, pregnant women generally decrease their level of physical training as pregnancy progresses [18,26], due to both the physical changes resulting from the pregnancy and a combination of social and psychological factors [17]. Enhanced psychological well-being has been shown in women who regularly exercise during pregnancy [16]. Additionally, a study evaluating physical activity at six weeks post partum found indications of physical and psychological benefits in women who were able to exercise vigorously and avoid any decrease in their usual level of activity [27]. The literature on the frequency of PA post partum is limited, and to our knowledge no previous study has been published on the prevalence of PA among women who have experienced LBPP during pregnancy. The identification of determinants and outcomes of physical activity before, during, and after pregnancy has important implications for the development of strategies aimed at promoting a physically active lifestyle among women, and thus constitutes an important public health issue [18].

The main finding in this study was that almost half of women who had experienced LBPP during pregnancy reported regular leisure-time physical activity (PA) at six months after delivery. However, there was no significant difference in prevalence of PA between women with persistent LBPP and women with remission of LBPP. This study was a population-based, follow-up, cohort study of women who reported LBPP during pregnancy. As noted above, the literature on the general prevalence of post partum physical activity is limited. In one study of an American population, around 35% of the respondents reported vigorous exercise with a modal frequency of three times per week at six weeks post partum [27]. In the current study, among those respondents who reported current PA, the mean frequency of PA since start of PA was 3.4 PA events per week. In a Swedish population from 1995 (n = 2342), physical inactivity before, during, and after pregnancy was reported in 48% of women with BMI < 24 kg/m^2 ^and 58% of women with BMI ≥ 24 kg/m^2 ^[28]. In our study, four out of five women with BMI ≥ 30 reported pre-pregnancy PA, while three out of ten women with BMI ≥ 30 reported current PA. In a previous publication we have shown that persistent LBPP was associated with higher BMI [9].

The overall prevalence of 'PA at some period in life' previous to pregnancy was 82.2%. Almost half of these women reported current PA, and, interestingly, almost one in four women who had not reported pre-pregnancy PA declared current PA. The current study was not designed to investigate possible incentives for the initiation of post partum PA in women who did not previously practise PA; however, it is possible that the experience of LBPP during pregnancy may have influenced the women to begin PA in order to improve their health. Further, the level of PA post partum must be assessed in relation to the transitional period of parenthood (or extension of the family) which may induce problems within the family and could be considered to be a critical stage in life [29]. Support from partner, family, and friends has been found to be a significant factor in maintaining or increasing physical activity post partum [19]. The mean time of start of PA was 2.6 months after delivery; Figure 1 reveals a fairly stable increase in the cumulative prevalence of PA during the period under investigation. The level and the starting point of post partum PA should also be considered in relation to the puerperal period in which the maternal physiology returns to its pre-pregnancy state.

We have previously demonstrated that a higher number of years of PA prior to pregnancy decreases the risk of LBPP during pregnancy [21]. However, in univariate and multivariate logistic regression analyses in the current study, previous PA did not significantly influence the risk of persistent LBPP six months post partum. It is not possible to determine whether this lack of association is due to a too-small sample size or actually mirrors a non-causal situation; however, the (non-significant) calculated estimates did not indicate the possibility of a major effect. The protective effect of pre-pregnancy PA may be due to the elevation of a threshold which retards or prevents the development of LBPP. If this is in fact the case, then if the threshold is exceeded, the protective effect may be lost and the condition will develop in accordance with its own nature.

### Methodological considerations

Extensive discussion of the validity of the materials has been presented in a previous publication [9]. In summary, the non-respondents did not differ from the respondents with regard to maternal age, gestational age, birth weight, mode of delivery, total experience of the delivery, epidural or spinal anaesthesia during delivery, and pre-pregnancy or end-pregnancy BMI. We consider the data most probably representative for women with persistent LBPP after pregnancy. It is possible that the lack of associations in the current study may be due to the use of a blunt tool. The literature is very sparse in this research area; however, in a study which compared a self-report exercise diary with a pedometer, the exercise diary was concluded to be a quantifiable method for measuring levels of activity during pregnancy [30].

## Conclusion

Almost half of women who had experienced LBPP during pregnancy reported regular leisure-time physical activity (PA) at six months post partum. The number of years of pre-pregnancy PA did not influence the risk of persistent LBPP. Obesity was a risk factor for not practising PA at six months post partum. More studies are needed in this research field to evaluate the influence of previous and current PA on the risk of persistent LBPP at six months after pregnancy.

## Abbreviations

*Q1*: the first questionnaire after delivery; *Q2*: the second questionnaire at approximately six months after delivery; *SD*: standard deviation.

## Competing interests

The author declares that they have no competing interests.

## Authors' contributions

IM performed all parts of the study and the manuscript.

## Pre-publication history

The pre-publication history for this paper can be accessed here:



## Supplementary Material

Additional file 1 **Background and outcome factors.** Test for difference between groups (*t*-test for parametric data and Pearson's chi-square for categorical data). Click here for file

Additional file 2 **Odds ratios (OR) with 95% confidence intervals (95% CI) for persistent LBPP^a ^post partum in relation to specified variables in univariate and multivariate logistic regression analyses.** Click here for file

Additional file 3 **Test for difference between groups (*t*-test for parametrical data and Pearson's chi-square for categorical data).** Click here for file
