# Supplementary material for: Physical activity and persistent low back pain and pelvic pain post partum
Source: BMC Public Health. 2008 Dec 22;8:417. doi: 10.1186/1471-2458-8-417 (PMC2630950; doi:10.1186/1471-2458-8-417)
Supplement: Additional file 1 — Background and outcome factors. Test for difference between groups (t-test for parametric data and Pearson's chi-square for categorical data). [file 1471-2458-8-417-S1.doc]

Table 1 Background and outcome factors. Test for difference between groups (*t-*test for parametric data and Pearson’s chi-square for categorical data)

| **Variable** | **All**  **Subjects** | **No pain^1^** | **Recurrent pain (LBPP)^2^** | **Continuous pain (LBPP)^3^** | **P-value^a^** | | | **Non-respondents** | |
| --- | --- | --- | --- | --- | --- | --- | --- | --- | --- |
|  |  |  |  |  | **1 vs. 2** | **1 vs. 3** | **1 vs. 2+3** |  | **p-value^4^** |
| **Number of subjects** (%) | **464** (100.0) | **264** (56.9) | **168** (36.2) | **32** (6.9) |  |  |  | **175** |  |
| **Age in years at start of PA**^5^  **No** (range; SD) | **12.8**  **365** (3-40; 5.6) | **12.8**  **205** (4-34; 5.6) | **12.9**  **136** (3-40; 5.7) | **13.1**  **24** (5-24; 5.2) | 0.906 | 0.823 | 0.970 | **12.6**  **125** (5-30; 5.0) | 0.646 |
| **Pre-pregnancy PA**  - yes **N** (%)  - no **N** (%) | **461**  **379** (82.2)  **82** (17.8) | **263**  **212** (80.6)  **51** (19.4) | **166**  **141** (84.9)  **25** (15.1) | **32**  **26** (81.3)  **6** (18.8) | 0.253 | 0.931 | 0.300 | **171**  **132** (77.2)  **39** (22.8) | 0.154 |
| **Current PA**  - yes **N** (%)  - no **N** (%) | **463**  **206** (44.5)  **257** (55.5) | **264**  **118** (44.7)  **146** (55.3) | **167**  **71** (42.5)  **96** (57.5) | **32**  **17** (53.1)  **15** (46.9) | 0.657 | 0.366 | 0.919 |  |  |
| **Mean number of weekly events of current PA**  **N** (SD) | **3.4**  **206** 1.8) | **3.4**  **118** (1.7) | **3.2**  **71** (1.7) | **4.2**  **17** (1.9) | 0.419 | 0.071 | 0.934 |  |  |
| **Mean start of PA (months after delivery)**  **N** (SD) | **2.6**  **203** (1.6) | **2.6**  **117** (1.6) | **2.6**  **69** (1.6) | **2.1**  **17** (1.6) | 0.253 | 0.931 | 0.604 |  |  |

**^1^** ‘No pain’ denotes respondents reporting remission of LBPP

**^2^** ‘Recurrent pain’ denotes respondents reporting recurrent LBPP after pregnancy

**^3^** ‘Continuous pain’ denotes respondents reporting continuous LBPP after pregnancy

**^4^** Non-respondents vs. respondents

**^5^** PA = regular leisure-time physical activity
