# Supplementary material for: Physical activity and persistent low back pain and pelvic pain post partum
Source: BMC Public Health. 2008 Dec 22;8:417. doi: 10.1186/1471-2458-8-417 (PMC2630950; doi:10.1186/1471-2458-8-417)
Supplement: Additional file 2 — Odds ratios (OR) with 95% confidence intervals (95% CI) for persistent LBPPa post partum in relation to specified variables in univariate and multivariate logistic regression analyses. [file 1471-2458-8-417-S2.doc]

Table 2 Odds ratios (OR) with 95% confidence intervals (95% CI) for persistent LBPP^a^ post partum

in relation to specified variables in univariate and multivariate logistic regression analyses

| Variable | **Crude OR**  (n=368) | | **Adjusted for maternal age**  (n=368) | | **Adjusted for parity**  (n=368) | | **Adjusted for parity, and age at start of PA** (n=365) | |
| --- | --- | --- | --- | --- | --- | --- | --- | --- |
| **LBPP**: |  |  |  |  |  |  |  |  |
| ***Number of years of PA***^b^ | ***COR*** | 95% CI | ***OR*** | 95% CI | ***OR*** | 95% CI | ***OR*** | 95% CI |
| 1-5 years | 1.00 |  | 1.00 |  | 1.00 |  | 1.00 |  |
| 6-10 years | 0.88 | 0.49-1.57 | 0.91 | 0.50-1.65 | 0.84 | 0.47-1.51 | 0.87 | 0.47-1.60 |
| 11-15 years | 0.90 | 0.49-1.63 | 0.95 | 0.51-1.75 | 0.91 | 0.50-1.67 | 0.92 | 0.48-1.77 |
| 16-20 years | 1.18 | 0.62-2.25 | 1.19 | 0.61-2.32 | 1.18 | 0.61-2.25 | 1.15 | 0.55-2.39 |
| 21-38 years | 0.86 | 0.36-2.01 | 0.70 | 0.28-1.73 | 0.84 | 0.35-1.98 | 0.85 | 0.33-2.15 |

^a^ LBPP = low back pain and pelvic pain

^b^ PA = regular leisure-time physical activity
