# Supplementary material for: Physical activity and persistent low back pain and pelvic pain post partum
Source: BMC Public Health. 2008 Dec 22;8:417. doi: 10.1186/1471-2458-8-417 (PMC2630950; doi:10.1186/1471-2458-8-417)
Supplement: Additional file 3 — Test for difference between groups (t-test for parametrical data and Pearson's chi-square for categorical data). [file 1471-2458-8-417-S3.doc]

Table 3 Test for difference between groups (*t*-test for parametrical data and

Pearson’s chi-square for categorical data)

| **Variable** | **BMI**^a^ **< 30** | **BMI ≥ 30** | **P-value** |
| --- | --- | --- | --- |
| **Pre-pregnancy PA**^b^  N (%)  - yes  - no | **392**  326 (83.2)  66 (16.8) | **53**  42 (79.2)  11( 20.8) | 0.479 |
| **Current PA**  N (%)  - yes  - no | **394**  184 (46.7)  210 (53.3 | **53**  17 (32.1)  36 (67.9) | 0.044 |
| **Mean no of weekly events of current PA**  (N; SD) | **3.4**  (184; 1.8) | **3.3**  (17; 1.4) | 0.835 |
| **Mean start of PA (months after delivery)**  (N; SD) | **2.6**  (181; 1.6) | **3.0**  (17; 1.4) | 0.329 |

^a^ BMI = body mass index

^b^ PA = regular leisure-time physical activity
